# 

**Published:** 1826-04

**Authors:** 


					LITERARY NOTICE.
The Anatomy of the Brain, with a General View of the Nervous
System. By J. G. Spurzheim, M.D. of the Universities of Vienna
and Paris, and Licentiate of the Royal College of Physicians in London.
Translated from the unpublished French Manuscript, by R. Willis,
Member of the Royal College of Surgeons in London. One Vol. 8vo.
with Eleven Plates.
N. B. Mr. Battley's Note came loo late.
XVI.
BIBLIOGRAPHICAL RECORD;
OR,
Works received between the 15th of December, and the
15th of March, 1825.
j
Cal ToContV'lhutlons *owar<k ^ie Medical History of the Waters, and Medi-
Cheltenham. Containing Directions for drinking the
? ' "y John Fosbhoke, resident Surgeon at Cheltenham.
&c. better to Sir Astley Cooper, Bart. F.R.S., Surgeon to the King,
t?tnicai 0I1jeita'n_ Proceedings connected with the Establishment of an Ana-
Pi'ofesso ^ f Ur^ca^ School, at Guy's Hospital. By J. H. Green, F.R.S,
Surger l. Anatomy to the Royal Academy; Professor of Anatomy and
tal ? an(j j? t"e Royal College of Surgeons ; Surgeon to St. Thomas's Hospi-
PP* 75 l8^5tUl er ?n Anatomy and Surgery at that Hospital. London, 8vo,
tlle CohimV/.tUre delivered at the Opening of the Medical Department of
1?e^vAll , lan College, in the District of Columbia, 1825. By Thomas
1825. ' Professor of Anatomy and Physiology. Washington City,
?f inediculV ?mtr?(*ucto.ry lecture gives an exhilarating picture of the progress
Vor T\Terxt an^l*ter<*ture in the United States.
?L- IV. No. 8. 2 Q
594 Bibliographical Record ; or [April
4- A Treatise on the Physical and Medical Treatment of Children.
William P. Dewees, M.D. &c. 8vo. pp. 496. Philadelphia, 1825.
5. An Essay on the remote and proximate Causes of Phthisis Pulmonale
being the Essay to which the Prize was adjudged for the year 1825, by the
New York State Medical Society. By Andkew Hamersley, M.D. Svo-
pp. 54. Philadelphia, 1825.
very creditable Essay, and well deserving the prize which ivas
judged to it.   .
6'. An Essay on the application of the Lunar Caustic in the cure of
. Wounds and Ulcers. By John Higginbottom, (Nottingham) Member 0
the Royal College of Surgeons of London. 8vo. pp. 147- Longman & Co-
January, 1826.
7- The Edinburgh New Dispensatory: Containing, 1. The Elements
Pharmacy. II. The Materia Medica ; or, the Natural, Chemical, and Me"
dical History of the Substances employed in Medicine. III. The Pharro<v
ceutical Preparations and Compositions ; including translations of the Lo?'
don Pharmacopoeia of 1825, of the Edinburgh Pharmacopoeia of 1817> aI1
of the Dublin Pharmacopoeia of 1807, with ample Commentaries and nui?e*
rous Tables. Eleventh Edition much improved. By Andkew Dunca>'?
jun. M.D. &c. Edinburgh, 1826". 8vo. pp. 9G0. Longman and Co. Lo11'
don, 1826.
fcf" In this Edition of a highly popular ivork, every department is broug^
to a level with the existing state of our knowledge, and, therefore, the alt^'a
tions and improvements are both numerous and important?more so, we belicl'e'
than in any preceding Edition. To make room for some of these improve
ments, the Author has wisely left out the " Epitome of Chemistry," note s'1'
perseded by various works on that delightful science. Still, the volume is
creased considerably in size. Dr. Duncan has greatly extended the princip^
of Pharmacy, as being a subject of primary importance, and he has added m(flV
tables connected with Pharmaceutic Chemistry, which ivill be found very >lS^
ful. The Materia Medica has been increased by the introduction of sevefj
new articles?while the chemical history of simples has been improved by
recent analyses of the more active vegetable substances. Their medical histoff
has also received many corrections and modifications from the clinical obsefva
tions of himself and other practitioners. From this short notice, it will "
readily anticipated that the present work is a most valuable acquisition to t
library of every practitioner, from the highest to the loivest in the professio
Of Dr. Duncan's ability to execute such a task, ivith credit to himself and &
vantage to the public, no one could doubt; and the present Edition afforl
ample proof that his ability was exerted to the utmost.  ^
8. Observations on the Efficacy of White Mustard Seed ; with aparticu^
view to its recommendation as a means of augmenting the beneficial use
the Cheltenham Waters. By Charles Turner Cooke, Consulting a'1
Operating Surgeon, Cheltenham. 8vo. sewed, pp. 87- January, 1826.
(fdj* This is jioio a fashionable remedy, and, being an innocent one, ^
fashionable mania for taking it cannot be productive of those evils ivhich a
company the indiscriminate use, or rather abuse, of more active nostrum .
After a short introduction, almost the whole of the body of the work is a WeZ.Cf
transcript of Dr. Johnson's Essay on Civic Life, Sedentary Habits, 9
There is an acknowledgment in the preface, but this is not sufficient in litefa *
justice, for verbatim transcripts. Dr. Johnson, however, has no wish to cQl
plain ; but the transaction is hardly fair towards the public.
1826] j'Porks received for Review since last Quarter. 595
Practi^ Treatise on the Diseases of the Eye ?, including the Doctrines and
Pi'ofes?e d ^le mosfc eminent Modern Surgeons ; and particularly those of
more p? eer* By George Frick, M.D. Ophthalmic Surgeon totheBalti-
bANk vfnera^ dispensary. A new Edition, with notes. By Richard Wel-
Chiru' m^er of the Royal College of Surgeons, and of the Medical and
30S Urf qCo^ Society of London. With an Engraving. London, 8vo. pp.
? ??ci26.
pracr an exceedingly useful compendium of ophthalmic doctrines and
deriv ?fi' lnr^u^'lnS all the more recent discoveries, and blending the knowledge
crtiin Jrom Continental practitioners with that of our own surgeons of
c>lce- JVe hope to notice the work more fully in our next Number.
as " Observations on the Lepra Arabum, or, Elephantiasis of the Greeks,
oj aPPears in India. By Whitelaw Ainslie, M.D. M.R. A.S. 4to. pp
1826 ) 1 ?m ^St Transactions ?f Royal Asiatic Society
11.
Revue Medicale, &c. for December, 1825.
Pli-p ^ System of Phrenology. By George Coombe, late President of the
Lornl^0 ??ical Society. Second Edition. 8vo. pp. 566. Edinburgh and
0n> 1825. With a Plate, Price 12s. boards.
vo(u 'f independently of its importance as a " System of Phrenology," this
one of P,resents verV high claims to attention, in being, as ice humbly conceive,
ever / fincst examples of philosophical reasoning and instruction that has
c?ncifeen su^nitted to the judgment of mankind. Throughout, it is modest,
induct?' PcrsP}c}lous '? 011 all questions, it exhibits premises fairly stated and
fats n?nS S^imately drawn : it abounds with good feeling and taste, mani-
throni (lr^c.n*' but temperate, zeal for truth, and has a rich glow of philan-
its I charms, without fascinating the mind: hence, we conclude that
decid.iienCeS 011 sc*ence and morals, will prove as extensive as they must be
cvidi beneficial.?In our next Number we shall place before our readers the
ticul ^ ?n w^Llc^L we have founded these sentiments, in a comprehensive analy-
13. Myology, illustrated by Plates. In four parts. Part the fiist, Mus-
es of the Anterior and Posterior part of the Thigh, Leg, and Foo . e i-
cated to the Governors of the Middlesex Hospital. By E. W. Tuson, House-
C5Urgeon. Folio, 1826.
This is the closest imitation of Nature ivhich can possibly be effected
0n Paper. Each muscle, coloured and proportioned, can be raised, as in dis-
\cction, exposing layer after layer, the various strata, till we come to the bone.
i,le plan is exceedingly ingenious, and the execution highly meritorious.
I80f" The Edinb"rgh Medical and Surgical Journal, No. 86, for January,
-o. 8vo. pp. 244. In exchange.
I8<>f' The Edlnl)ul'gh Journal of Medical Science, &c. No. 1, for January
J~~ ? 8vo. pp. 248. In exchange.  ___________
ui.L6*,Medical and Surgical Cases; selected during a practice of thuty-
Dd j#vfrs' Edward Sutleffe, Queen-Street, London. ? ?
? London, February, 1826.
596 Bibliographical Record. [April
17. A Critical Enquiry into the Ancient and Modern Method of curing
Diseases in the Urethra and Bladder; and of the successful practice of Vesic#
Lotura, for the cure of diseased Bladders. The whole illustrated by a great
variety of cases. By Jesse Foot, Surgeon. Carefully revised and cor-
rected. By Je^se Foot, jun. Member of the Royal College of Surgeons-
Eighth Edition. London, 8vo. pp. 201, with ablate. 1826.
18. Dissertatio Physiologica Inauguralis de Absorbendi Functione. By
Thomas Hodgkin, M.D. 8vo. pp. 78. Edinburgh, 1823.
This is the most erudite dissertation on the subject of absorption which
we have seen, and does great credit to Dr. Hodgkin, who, in this and other
countries, has laid in a stock of practical information, in addition to an excel'
lent elementary education, which, we venture to prognosticate, will, one day*
give him eminence in his profession.
19. An Experimental Inquiry into the Laws of the Vital Functions. W
A. P. W. Philip, M.D. F.R.S. E. in part republished by permission of th?
President of the Royal Society, from the Philosophical Transactions of 18'^
17 and 22, with the Report of the Institute of France on the Experiments
Legallois, and Observations on that Report. Third Edition, addressed to
the Scientific Public. 8vo. pp. 33.9. Underwoods, February, 1826.
?5^f=* In our next.
20. A System of Anatomical Plates ; accompanied with descriptions, and
Physiological, Pathological, and Surgical Observations. By John Lizab*>
F.R.S. E. &c. Part IX?the Organs of Sense, &c. 8vo. pp. 209 of Letter'
press, Ten Plates, demi-folio. February, 1826.
The increasing difficulties thrown in the way of Anatomy (which
Mr. Lizars feelingly deplores) render this work still more important and *ise'
ful than at any former period. The spirit and beauty of the plates are kept
in this fasciculus, and the letter-press contains some very important patholog1'
cal observations.
21. An Elementary Compendium of Physiology; for the use of Students-
By F. Magendie, M.D. &c. translated from the French, with copious notes*
tables, and illustrations. By E. Milligan, M.D. Fellow of the Royal Col'
lege of Surgeons, &c. Second Edition greatly enlarged. 8vo, pp. 61"*
March, 1826.
J?0 This Edition is enriched by a large mass of important notes by tk*
translator?*and is altogether a most valuable elementary work of physiology'
22. A Plate of the Eye, engraved by Stewart, from a drawing by A-
Rowlands, after the plates of Zinn and Soemmerring, and used by
'Mayo in his lecture-room, Berwick-Street, Soho. Published by Burgess
Hill, Great Windmill-Street. 1826.
This plate, which is on a very large scale, contains three figures, W?s\
beautifully and accurately representing the various coats, humours, vessels,
nerves of the organ of sight. It is, without comparison, the first thing of*
kind which has appeared in this country.
23. The Medical Recorder, &c. No, 33, (quarterly series) for Janus'*)''
J 8J6, Jn exchange.
1826]
Intelligence, &c. 597
aerico'f*let*S>ew ^ork Medical and Physical Journal, No. 16, (quarterly
' or 'ast quarter of 1825. In exchange.
*0fh?*E?Authors and publishers will readily appreciate the importance
st ^Vlng their works recorded, with full length titles, on this list, which
far -aS a PerPetual advertisement so long as the Journal lasts, and as
th *,* exten^s- The republication of the Journal in America enhances
jj e ^vantages of the Bibliographical Record, on which 110 work can
ce entered, unless transmitted, free of expense, to the Editor, under sealed
cone1 (j16 Pu^^s^ers? or in any other way mos^ convenient to the parties
ce/'- ^lC Bibliographical Record closes on the 15th of the month pre-
Plication, all JVorks received after that date necessarily stand over
next quarter.
$00
Additional Subscribers since last Quarter.
A.
Archer, Mr.Chs. Student, London.
B.
Benson, Mr. Charles, Uppingham.
Berenger, Mr. Surgeon, to Mexico.
Boetticher, Mr. Richmond.
Burgess, Dr.Castle-street, Hastings.
Burnie, Dr.
c:
Caldwell, Dr. United States.
Cox, Dr. (residence not stated)
F.
Fulton, Dr. Leamington, War-
wickshire.
G.
Gellatly, Dr. Member of Royal
College of Surgeons, London,
London-road, Southwark.
M.
Meadows, Mr. Thomas, Com-
pany's Marine Service.
Mendur, Dr. William Hall, of the
Faculty of Physicians and Sur-
geons, Glasgow,
Mills, Mr. James, Edinburgh.
N* ' .
Noakes, Thos. Chemist, &c. Ox-
ford-street.
O.
O'Connell, Dennis, M.D. Mill-
street, Cork.
O'Gorman, Mr. Surgeon, Cadiz.
R.
Rolland, Patrick, Esq. Surgeon,
Montrose.
S. _
Serjeant, Mr. William, New Or-
leans.
Sweeney, Dr. Staff Surgeon.
T.
Telfair, Chs. Esq. Honble. Com-
pany's Service.
Turton, Thomas, Esq. Howden.
W.
Whitmore, William, Esq.
Wilson, Mr. John, Surgeon, Clerk-
street, Edinburgh.
TO CORRESPONDENTS.
The letter of Medicus on Bloodletting and the Exhibition of Mercury will
be applied to use in our next. Dr. Fulton's communication has been re-
ceived, and will be noticed in the succeeding Number of this Journal. The
hints of Brutus are applied under the proper head, in the present Periscope,
as he will readily perceive. We are particularly obliged to Mentor, and
solicit a continuance of his favours. We have been obliged still longer to
postpone several of Dr. H?s' papers from continental writings, but they
will certainly appear in our next. The same apology is due to Dr. ? of
? G.?The extremely interesting case of empyema, successfully treated by
the operation of paracentesis, by Mr. Jovvett, of Nottingham, alluded to in
page 564 of this Number, we shall give, at our own expence, in the extra-
limites department of our next. The present Number has so much over-run
the regular limits that we were obliged to postpone its publication.
Want of room prevents us from replying to several queries, and acknow-
ledging several communications.?18th, March, 1826.
IN DE X
A.
Abdomen, pressure on  521
Abdomen, serious wounds of;
cases    547
Abdomen, state of it, after rup-
tured uterus  23
Abdominal tumour, 400 leeches
applied! 201
Abortion, from compressing vari-
cose veins  245
Absorbents, the effects of iodine
upon them  ^
Adulteration of wines  313
Aggregation of bone, thoughts on 58
Agile, interesting case of  131
Ainslie, Dr. on elephantiasis.... 443
Amaurosis, sympathetic, with dis-
oidered bowels  269
Amaurosis, Dr. Belcher's case of.. 572
Amputation by the flap  237
Anatomy, Monro's Elements of.. 52
Aneurism cured by aneurism.... 533
Aneurism, aortic, case of.  200
Angina pectoris, mistaken notion
of its cause   372
Angina pectoris, marked case of 497
Annesley, Mr. on the diseases of
India    88
Annesley} Mr. on the effects of ca-
lomel   328
Antacids, specific in cholera mor-
bus .    259
Antiquity of the Caesarian section 474
poplexy, its severe forms  366
poplexy, sanguineous; a most
astounding case  267
rachnitis, case of  210
?"nistrong, Dr. his letter to the
College  273
ferial action not perceptible for
-4 hours..   102
rteritis, Mr. Howel's case of.... 193
?"tificial anus, ?Dr. Martland's
. rase  233
prides, death from  287
Scites, its causes and treatment 411
Us.cultation, Bennett's The-
sis on  449
yre, Dr. on dropsy   406
^ his observations on
bSSiTv"?????? 364
\ ?ur- "is opinions as to suc-
Ra;iiSS m niedicine  117
rf 'c> bis works, by Ward-
Barry f)V V "": "" 115
i?,. r* "is views ot the circu-
Uon  488
I
Barry, Dr. his treatment of poi-
soned wounds    518
Bayle, M. his views of the causes
of madness....     185
Begbie, Dr. his ease of supposed
fractured cervix femoris 551
Bell, Dr. his unfortunate case.... 74
Biliary secretion, Dr. Simon's ex-
periments on .  488
Bleeding successful in a case of ir-
ritative fever  70
Bleeding in irritative fever, con-
sidered  77
Bleeding, when indicated  Ill
Bleeding, late in fever, its good ef-
fects  132
Bleeding in dropsy  137
Blood, its state in epidemic Indian
cholera     93
Bone formed in different organs ;
case of  119
Bones, their thickness?on what
dependent ?   57
Bostock, Dr. on the mercurial sa-
liva   400
Brain, symptoms of irritability of
the  436
Brain, Dr. Gregory's case of hae-
matoid tumour of the 523
Brain, its consistence and state in -*?
inflammation  194
Brain, ulcer of, Dr. Scoutteten's
case of.    208
Bronchocele, its etiology  2
Bryce, Mr. his new syphonic sto-
mach-pump   264
Burnett, Dr. his curious case of
epilepsy  502
Burnt sponge, objections to its use 6
Butter, Dr. on the dock-yard fever 66
C.
Casarian section successful  573
Ca;sarian section, Drs. Mansfield,
Schenk, and Meyer on  473
CtEsarian section, modes of, re-
viewed      476
Calomel?versus prejudice.. 329
Calomel, its action on the secre-
tions    334
Calomel, prejudices 3gainst...... 253
Camphor and hyoscyamus in go-
norrhoea  ........ 537
Cantharides, a case of poisoning
by   272
Caries of the vertebra;, remarka-
ble case of.  131
Carotid, one obliterated, Dr. Bail-
lie's remarks ........  J19
INDEX,
Carotid tied beyond the aneurism 528
Cartwright, Mr. his Essay on sy-
philis    328
Catalepsy,astonishing case of.... 201
Cataract, early removal of, by ab-
sorption     234
Cautery, actual, its virtues  219
Cellular texture, diffuse inflam-
mation of  255
Cerebellum, case of hydatid of.. 525
Cervix femoris ; supposed case of
fracture  551
Cliarit^, Reports from that hospi-
tal   196
Cheap anatomy and its conse-
quences   550
Chlorate of soda, Dr. Darling on 574
Cholera morbus, Dr. Ainslie on.. 258
Chorea, Dr. Venables on  260
Chorea, iodine in  9
Cicatrices of burns, excision of.. 553
Cicatrization, a new mode of in-
ducing  283
Circulation in the veins, Dr. Bar-
ry's Theory  488
Clinical instruction in France and
Germany  216
Colchicum in gout, Dr. Scuda-
more on     44
Colchicum, various preparations
of.  46
Colica pictonum, counter-irrita-
tion in  282
Colica pictonum and" dry belly-
ache," distinguished   251
Colica pictonum, salivation in.. 198
College of Surgeons and its Mem-
bers  581
Compact (Edema," M. Leger's
Memoir on  205
Compliments from our Gallic
neighbours    284
Concretions in the intestines, M.
Langier on .  303
Concussion, case of   555
Constitutional irritation, ratio-
nale of.    105
Consumption in France, fearful
prevalence of.    493
Contraction of the chest, some in-
stances of..  563
Cooper, Sir A. his lectures on sur-
gery    98
Cooper, Sir A. his lectures  397
Craniotomy, directions for its perr
formance  .V  160
Croton tiglium, new preparation
of  573
Croton oil in West Indian yellow
fever    263
Croup, Mr. Mackenzie on,? ?? , 261
Croup, Mr. Pretty on   590
Crystalline lens, its reproduction
asserted   ,... 303
Cupping glasses to poisoned
wounds    589
D.
Davis, Dr. on operative midwifery 148
Davis, Dr. on the ergot of rye.... 276
Deafness, iodine in    13
Death from excessive depletion ;
melancholy case  355
Death after forceps operations.. 157
Decayed teeth, curious cases of ir-
ritation from    101
Delirium tremens, speculations on 37
Delirium tremens, cases of  38
Delivery, immediate, in ruptured
uterus  24
Determination to the brain, case
of  145
Diabetes, venesection ill  260
Diarrhoea, after severe labours.. 155
Dislocation and fracture of the cer-
vjx femoris  543
Dissecting, slovenly mode of, in
the French school.,    215
Dissection-wounds treated by lu-
nar caustic  383
Dissection-wounds, distinctions of 384
Dix, Mr. his case pf wounded ab-
domen  549
Doctors; shewing how at times
they disagree  441
Dropsy after haemorrhages  134
Dropsy, a curative process, case of 135
Dropsy, Drs. Ayre and Shearman
on  406
Dryden, Mr. his Report on the
Dock-yard fever   66
Ducasse, M, his expose of French
surgeons  550
Duodenum, not quite a second sto-
mach     422
Dyspepsia, mode of treatment of 43
Dysphagia, case of, cured by io-
dine    15
E.
Edinburgh Society; the subject of
their prize-essay  299
Effusion on the brain, recoveries
after     430
Egophony, description of;.46?.
Electricity in the atmosphere, M.
Pouillet on.        485
Electricity as a vital agent, Dr.
Edwards' researches on  283
Elephantiasis, Dr. Ainslie's paper
on   443
Elephantiasis, treatment of.. .... 443
index.
Elliotson, Dr. his case of rupture
of the stomach...  326
Embryo, Dr. Pockels on the hu-
man.....  575
Empyema, Dr. Hastings' paper on 557
Empyema, successful case of ope-
ration for  5G2
Epilepsy, Dr. Williams' case .... 259
Epileptic convulsions, Dr. Blake
on.  592
Ergot of rye, cases in which it was
tried.    276
Erysipelas, blood-letting in  269
Erysipelas, sulphate of quinine
very valuable in.    404
Eschars by lunar caustic, healing
sores, &c. by    380
Etiology of the " Dock-yard dis-
ease,"   85
Etiology of the epidemic Indian
cholera    94
Euphorbia lathyris, oil of, just im-
ported  301
Excision of piles, arguments pro
et con   362
Expectoration, various kinds of.. 465
Experiments with calomel in large
doses ..  381
Extra-uterine conception, Dr.
Wishart's case...  280
Extra-uterine and super-fcetation 285
F.
Face-presentations considered .. 154
Fat, not formed in the great in-
testines   31
Fear, its influence on the human
frame  110
Fever, symptomatic treatment of 377
Fever, a bugbear to the French
physicians   570
Fistula lacrymalis, success of io-
dine in  11
Forceps, long, remarks on their
use, &c    149
Forceps, long, Dr. Davis's modifi-
cation of the  153
Forceps' operations, treatment af-
ter them  156
Formation of bone ; Monro's opi-
nion    56
Formula of a powerful tonic.... 374
Fracture of the patella and neck
of the femur  221
Fracture of the skull, with depres-
sion, Dr. Corban's case...... 241
France and Germany, their sur-
gery compared  213
French professors, scandalous oc-
currences amongst    ?83
Fungus melanodes, Mr. Wardrop's
account of...  122
Fungus of the uterus extirpated
by ligature  544
G.
Gangrene of the foot, Mr. Cheva-
lier's ease    324
Gangrene, treatment of  402
Gangrene of the lungs, M. Bouil-
laud's paper on  498
Gastrotomy in ruptured uterus,
case of.  26
Gastrotomy, a successful instance
of  285
Ghosts at Wakefield, with specu-
lations thereon  490
Globus antiperistalticus, M.Troi-
let on  510
Gonorrhoea, Mr. B. Bell's views of 535
Good, Dr. his study of medicine 387
Gout flying to the stomach, curi-
ous case  42
Gout, ancient treatment of  46
Gout, Dr. Scudamore's mode of
treating  48
Gout of the stomach, fatal case of 142
Granville, Dr. his case of indiges-
tion   566
Guthrie, Mr. on fractures of the
femur  540
H.
Haemoptysis, a forerunner of con-
sumption   495
Haemoptysis, large doses of nitre in 211
Haemorrhoidal excrescences, Mr.
Kirby on  358
Hall, Dr. Marshall, his Medical
Essays     341
Hastings, Dr. on empyema.  557
Hearing and speech restored; an
instance   .  138
Hectic, relieved on the appear-
ance of dropsy  133
Henderson, Dr. his History of an-
cient and modem wines  305
Hepatic ileus, Dr. Musgrave's pa-
per on  248
Hereditary nature of bronclioeele 4
Hernia, delay in operating for.. 233
Hernia, strangulated, case of.... 550
Higginbottom, Mr. his Essay on
lunar caustic  372
Hospital physicians and surgeons
their duty  197
Hotel Dieu?M. Recamier's Re-
ports  209
Howship, Mr. on indigestion.... 34
Hydatid formations, Dr. Bailey's
cases of  ...... 524
INDEX.
Hydrocephalus, instance of.... 145
Hydrocephalus tapped, Mr. Sym's
case  236
Hydrocephalus cured by pressure,
supposed case of.  262
Hydrocephalus interims, nature
and treatment of.  408
Hydrophobia  263
Hydrophobia, preventions of.... 265
Hydrophobia spontaneous, Dr.
Whymper's case of.  518
Hypcrtrophia cerebri, case of.... 523
Hypertrophies of the heart, real
and apparent  199
I.
Incarcerated intestine, fatal adhe-
sion of its surfaces  231
Indian diseases, Mr. Annesley on 88
Indigestion, Mr. Honship on.... 34
Indigestion, a fashionable case of 566
Inflammation, gouty, how treated 51
Inflammation, abdominal, in irri-
tative fever.   74
Inflammation, irritable, Sir A.
Cooper's definition of  110
Inflammation of the membranes
of the brain  187
Inflammation of the liver, &c. re-
markable case of  520
Injuries of the head, insidious na-
ture of  242
Insensibility of the retina........ 303
Intestinal irritation, its symptoms 343
Intestine small, loss of upwards of
4 feet of      30
Intermittents, M. Recamier's phi-
losophical treatment of  209
Internal abscess, an interesting
case..  286
Iodine, Dr. Manson's researches
on ...     1
Iodine, formula for a tincture of it 4
Iodine, its powers in a case of pal-
sy  7
Iritis, closure of the pupil in, Mr.
Mackenzie's Theory  239
Irregular determination, causes of
&c   130
Irritative fever, Messrs. flutter
and Tripe on  63
Irritative fever, interesting case of 68
Irritation, Sir A. Cooper's views
of  100
Irritation, urethral, very remar-
kable case of  102
Irritation, treatment of  108
Irritable ulcer, application for.. 401
J.
James, Mr. on excision of cica-
trices    553
Johnson, Dr. bis reply to Dr.Good's
criticisms
394
John Bull, no leper   447
Jowett, Mr. his successful case of
operation lor empyema...... 56'4
K.
Key, Mr. Aston, his case of liga-
ture of the subclavian  319
Kirby, Mr. his extirpation of the
parotid  546
Knee-joint wounded, treatment of 399
L.
Lacerations of the uterus, Dr.
M'Keever on    19
Lacerations of the uterus, symp-
toms of > .. .. 21
Laennec, M. his Hospital Reports 97
Laennec, M. his Hospital Reports 569
Laryngeal phthisis, herring-milt
not always a cure for  566
Laudanum, a case of poisoning by 270
Lead suggested in aneurismal dis-
eases   200
Lectures of Sir A. Cooper ou sur- -
gery  98
Leeches, porter a good whet for.. 285
Lepra arabum, its symptoms and
progress    444
Ligature on varicose veins, its suc-
cess in France....  247
Ligatures beyond the aneurism ;
Deschamps and Sir A. Cooper's
cases   529
Liniment, iodine, formula for it.. 5
Litharge, in wines, test of. 313
Lithotomy, haemorrhage in...... 538
Liver, state of, in gout   45
Longitudinal sinus, varicose con-
dition of.  514
Loss of blood, effects of  352
Louis, M. his interesting paper 011
phthisis  492
Lues , Mr. Hunter's dicta errone-
ous   392
Lunar caustic, Mr. Higginbot-
tom's Essay on  379
Lungs, deplorable tendency of to
disease   494
M.
Mackenzie, Mr. 011 closure of the
pupil in iritis  239
Mackenzie, Mr- 6n Croup  2b" 1
Manson, Dr. on the effects of io-
dine   I
Mansfield, Ur. on the antiquity of
the Ca;sarian section........ 473
Marrow, spinal, cases of destruc-
tion of it  181
Medical Essays, by Dr. Marshall
Hall  341
INDEX.
Medicine, its rank and observance
in society...  127
Medico-Chirurgical Transactions,
vol. XII  317
Meningitis, chronic, fatal case of 146
Mental alienation, Mr. Baylc's
views of  185
Mercury in " dry belly-ache.".... 253
Mercury, as a preventive of hy-
drophobia     266
Mercury, its physiological effects
on the system  491
Meyer, Dr. his successful case of
Caesarian section    481
Milky blood  591
M'Keever, Dr. on laceration of tha
uterus  19
Monro, Dr. his Elements of Ana-
tomy    52
Mucous membrane, adhesive in-
flammation of  397
Mussrrave, Dr. on " dry belly-
ache."    248
Myology, Dr. Monro's nomencla-
ture  65
Mysterious case of Henry Hyde,
Esq   527
N.
Nails " growing in," Dupuytren's
operation for removing  225
Nitrate of silver, an application in
croup   261
O.
cObservations on medicine, by the
late Dr. Baillie  364
Obstetric Society; notice of it.. 302
.(Esophagus, constriction of, in
hysteria    35
Olfactories, not the nerves of smell 303
Opacities of the cornea, Dupuy-
tren's panacea for   229
Operative midwifery, Dr. Davison 148
Ophthalmic surgery in France,
quackery of  226
Otitis, Dr. Kennedy on  591
Ovarian dropsy, its fatality  418
P.
Pain, effect of, on the powers of
the heart     528
Painful affections of the stomach. 41
Palpitations of the heart  370
Paralysis, iodine in    7
Parotid Gland, case of extirpation
of   546
Parry, Caleb, his posthumous
works    124
Patella, rupture of its ligament.. 239
Patients, their habits to be con-
sidered    106
Pathology of Indian cholera .... 91
Pathology of delirium tremens.. 38
Pathology of " compact oedema" 206
Pelvis, measurement of    59
Percussion and the stethoscope,
Dr. Ryland on  449
Perforation of the stomach, cu-
rious case of  ?16
Peripneumony not exactly similar
to hydrocephalus ..... 443
Petechiae htemorrhagicae, case of 139
Phlebectasia ; fatal case  507
Phlebectasia, Sir A. Cooper's opi-
nions on     402
Phlebitis, some fatal cases of.... 513
Phlebitis, Messrs. Bouillaud and
Ribes on  504
Phthisis, state of the expectoration
in    469
Phthisis pulmonalis cured   195
Phthisis, the exhalations of marine
plants in..   199
Pleuritis, instructive case of.... 139
Poisoning by laudanum, an in-
stance of ...?  270
Poisoning by cantharides, a case of 272
Portending symptoms, of rupture
of the uterus....v.  23
Pouillet, M. on atmospheric elec-
tricity  485
Premature labour, operation for
inducing    158
Pressure, its effects on bone .... 58
Prevention of gout  49
Prolapsed uterus extirpated .... 580
Ptyalisin, chlorate of soda in.... 574
Pulse, remarkable state of, in a
case of epilepsy   502
Purgatives, objections to, in cho-
rea    10
Purgatives iu irritability of the
brain  438
R.
lldlei, varieties of, with their
causes   456
Reaction after depletion, descrip-
tion of  353
Respiration, natural and morbid
states of....    451
Rheumatism, acute, bark in .... . 140
Rheumatism and gout identified! 141
Ribes, M. on inflammation of
veins      510
Robert, the Bruce, aecount of his
remains  57
Roux, M. on staphyloraphia .... 533
Ruptured uterus, case of recovery
after     29
Rupture of the uterus, long for-
ceps iu   150
Rutter, Dr. his reclamation .... 300
INDEX.
s.
Saliva, state of in mercurialization 490
Sanguineous apoplexy, a most
marvellous case of  267
Schenk's case of Caesariau section 478
Scrophula, cases of, treated by
iodine    11
Scudamore, Dr. on colchicum in ,
gout ........  44
Sea-sickness, as a methodusmedendi 36
Serum, in dropsical uterine ; Dr.
Well's statement   .... 407
Shaw, Mr. on lithotomy  540
Shearman, Dr. on water on the
brain  432
Snell, Mr. on obturateurs 598
Spinal marrow, case of great des-
traction of.     180
Spurious CcBsarian Section, re-
marks on the   25
Staphyloraphia....   533
Stethoscope, diagnostic powers of 571
Stevenson, Mr. on the absorbent
practice in cataract.......... 234
Stimulants, in irritative fever.... 87
Stokes, Dr. on the use of the
stethoscope    449
Stomach-pump, Mr. Bryce's new
one....    264
Stomach; prevalent affections of
this organ  372
Study of medicine; new edition., 387
Subclavian tied, Mr. Key's suc-
cessful case  321
Surgeons ; Dr. Armstrong's letter
to the College    273
Surgery, parallel of French and
German    213
Symphysis pubis, division of, in
labour    159
T.
Taste, horrible depravation of.. 282
Tetanus, a successful case  284
Tetanus, a successful case  285
Thigh-bone, Mr. Guthrie on frac-
tured neck of   540
Tonics, their good effects in am-
aurosis     572
Transfusion of blood, successful
cases   279
Transfusion of blood, Mr. Double-
day'^ case ..........  283
Traumatic tetanus in the horse.. 256
Traumatic tetanus cured, a case 267
Travers, Mr. on wounds of the
belly   548
Trephine, used for epileptic con-
vulsions  ,  592
Tripe, Mr. his two cases of " dock
yard fever."  78
Troilet, M, his paper on globus
antiperistalticus   519
Tumour iu the anterior mediasti-
num, curious case  323
Tumours, Dr. Good's hypothesis of
their growth, ike  389
U.
Ulcer on the surface of the brain,
case of     207
Ulcer, an odd travelling one.... 241
Ulceration of the stomach, Dr. El-
liotson's case  326
Urethrovaginal fistula cured.... 549
Uterine haemorrhage, plugging of
the uterus inr  278
Uterus ; fungus of, extirpated by
ligature   544
Uterus, Dr. M'Keever on lacera-
tions of it    19
V.
Varicose veins, Dr. Briquet's Me-
moir on...   245
Vascular excitement and inflam-
mation, contrasted  435
Veins, several eases of inflamma-
tion of the     504
Velpeau, M. on the spinal marrow 302
Venesection in irritative fever.... 70
Venesection in cholera  96
Venesection misapplied, a case of 345
Venous pulsation, strange instance
of.  92
Vertebral column, easy mode of
opening it  18
Vesical ulceration cured, Dr.
Crowther's case   573
Vesico-vaginal apertures, lunar
caustic in    281
Vesicula erythroides, discovery of
the  576
Visceral derangement leading to
hydrencephalus  423
Wardrop, Mr. his edition of Dr.
Baillie's works.  115
Wardrop, Mr. his case of ligature
beyond the aneurism  528
Water, filthy state of, in the me-
tropolis   307
Watery state of the blood in deli-
rium tremens  40
Weiss, Mr. his reply to Dr. Davis S95
White swelling, employment of
iodine in   17
Wines, Dr. Henderson's history of 305
Wines, their comparative virtues
and strength  314
Yeats, Dr. on hydrencephalus .. 406
Yellow fever, very interesting
case.,. ? 526

				

